# Effectiveness of virtual reality-based vestibular rehabilitation in patients with peripheral vestibular hypofunction

**DOI:** 10.55730/1300-0144.5545

**Published:** 2022-09-12

**Authors:** Yuşa BAŞOĞLU, M. Bülent ŞERBETÇİOĞLU, İlayda ÇELİK, Hasan DEMİRHAN

**Affiliations:** 1 Department of Audiology, Faculty of Health Sciences, İstanbul Medipol University, İstanbul, Turkey; 2 Department of Speech and Language Therapy, Faculty of Health Sciences, İstanbul Medipol University, İstanbul, Turkey; 3 Department of Otorhinolaryngology, Faculty of Medicine, İstanbul Medipol University, İstanbul, Turkey

**Keywords:** Vestibular rehabilitation therapy, virtual reality, vestibular hypofunction

## Abstract

**Background/aim:**

The rehabilitation of classical peripheral vestibular disorders is long and costly. Recently, interactive systems based on virtual reality (VR) technology have reduced the cost of vestibular rehabilitation therapy (VRT) and made the process more enjoyable. This study aims to investigate the effects of VR-based VRT in patients diagnosed with peripheral vestibular hypofunction (PVH).

**Materials and methods:**

In this study, a VR-based VRT program that utilized Sony Playstation®4 VR Head Mounted Display was applied to 25 patients (between 18–60) diagnosed with PVH. PVH was diagnosed by evaluating the patients’ clinical histories, the findings in the “Micromedical Technologies VisualEyes Spectrum” videonystagmography (VNG) and the “Micromedical Aqua Stim” model bithermal water caloric tests. VR-based VRT program was applied to the patients for 4 weeks, 2 sessions per week, 8 sessions in total. Each session lasted around 30 to 40 min. All patients underwent the Dizziness Handicap Inventory (DHI), Sensory Organization Test (SOT), Adaptation Test (ADT), Limits of Stability (LOS), and Rhythmic Weight Shift (RWS) before, after, and 8-week follow-up of the VRT program. In addition, the Cybersickness Survey was applied to the patients at the end of the VR-based VRT session every week.

**Results:**

The DHI mean scores of the patients were 54.60, 19.20, and 16.84, respectively, before, just after, and at the 8-week follow-up VRT (p < 0.001). The mean SOT composite score of the patients was obtained as 58.08 before VRT; 77.16 after VRT and 76.40 at 8-week after VRT (p < 0.000). On the other hand, the values in the ‘movement velocity’ and “direction control” parameters of the patients in LOS and RWS showed a significant improvement after VRT compared to before VRT (p < 0.000). From before VRT to 8 weeks after VRT, the patient’s oscillation averages in the ‘toes up’ and ‘toes down’ positions in ADT reduced progressively (p < 0.000).

**Conclusion:**

This study demonstrates that implementing a VR-based VRT protocol may be an efficient option to improve posture stability and the quality of life in patients with PVH. In addition, VR-based vestibular rehabilitation therapy has shown to be effective for PVH patients in the mid-term.

## 1. Introduction

Vestibular hypofunction (VH) is also known in the literature as vestibulopathy, vestibular dysfunction, hyporeflexia, and vestibular loss [1,2]. Unilateral or bilateral vestibulopathy is a heterogeneous disease that causes typical symptoms in the peripheral and/or rarely the central vestibular system [3–4]. Dizziness, instability, oscillopsia, nausea, and impaired spatial orientation are the most common symptoms of peripheral vestibular hypofunction (PVH). VH has been shown to affect approximately 53 to 95 million adults in Europe and the United States [5]. These symptoms adversely affect the patients’ quality of life. Patients may not be able to perform daily duties during this period, and this reflects on society as a cost [6]. Ward et al. showed that people diagnosed with vestibular hypofunction may have increased the risk of falling 31 times as much. [7].

There are many treatment methods for patients diagnosed with PVH. These are medical, surgical, and VRT approaches. In cases of peripheral vestibular system diseases, if medical treatment and rehabilitation programs are ineffective and the patient becomes unable to do his/her job due to vertigo attacks and ends up with isolation from society, surgical options may be considered for the solution of the problem [8–10]. Pharmacological treatment is recommended only in the acute phase. A mainstay for treatment in those who do not recover is customized VRT.

Cawthorne and Cooksey first introduced the concept of VRT when they presented a vestibular exercise protocol to increase the gaze and postural stability of patients with vestibular loss [11,12]. Current vestibular rehabilitation is based on an exercise that typically includes a combination of four different exercise components to improve and develop the functional limitations caused by vestibular deficit: i) exercises to increase gaze stability, ii) habitual symptoms (including optokinetic) exercises, iii) exercises to improve balance and gait (balance and gait training), and iv) walking for endurance. The main principle of VRT is to provide or accelerate a natural vestibular healing process, which is attributed to the phenomenon of vestibular compensation [13–15]. It is provided through neuroplasticity mechanisms (adaptation, habituation, and substitution) [15]. In literature, VRT is accepted as a safe and effective intervention method for PVH [13–16]. The traditional vestibular rehabilitation program includes an exercise protocol that can take up to 20–40 min of exercise several times a day. Therefore, traditional VRT may be accepted as boring and monotonous to patients. Recently, a virtual reality system has been integrated with vestibular rehabilitation exercises. Compared to traditional vestibular rehabilitation methods, VR-based VRT allows a wide range of stimuli with greater specificity and offers sensory inputs of different difficulty levels to the patient in a safe, comfortable, and standardized environment.

VR is a technological system that enables people to interact in a virtual environment through various sensory channels such as vision, hearing, and touch. In recent years, VR systems have also been an effective therapy method in the vestibular rehabilitation field. Games involving adaptation, habituation, and substitution exercise components have proven to be an effective treatment method, especially in treating chronic imbalance because of vestibular disorders, gaze stability in PVH patients, exercise training, various disorientation of patients, and postural control [17,18]. VR-based VRT has shown a similar effect to traditional vestibular rehabilitation [19]. Nevertheless, unlike conventional treatments, low-cost VR technology creates fun environments by increasing users’ interest and motivation. This method may become more appropriate than static posturography, and it may be possible to apply it as home therapy to maintain vestibular compensation. Using objective and subjective evaluation methods, the aim of this study is to determine the effectiveness of VRT with VR-based attention, gaze, and posture exercises in patients with dizziness, vertigo, imbalance, oscillopsia, and functional limitations after peripheral vestibulopathy due to various reasons

## 2. Materials and methods

This study was carried out in the **İ**stanbul Medipol University Hospital Audiology Unit between August 2020 and May 2021. The study was approved by the “**İ**stanbul Medipol University Non-Interventional Clinical Studies Ethics Committee” on 24.09.2020 (10840098-772.02.-E.49736). All participants provided written informed consent.

### 2.1. Study population

Our study included 25 patients diagnosed with PVH, 15 women and 10 men, aged 18 to 65 (average age 44.40 ± 11.44). Patients were diagnosed according to the consensus of the Barany Society’s International Classification of Vestibular Disorders, based on the diagnostic criteria of PVH.

Each patient’s medical story, bedside tests, “Micromedical Technologies VisualEyes Spectrum” model VNG and “Micromedical Aqua Stim” model bithermal water caloric tests findings were used to diagnose PVH. The inclusion criteria of the research group are as follows: peripheral vestibular impairment approved by VNG as canal paresis with directional preponderance, ability to adapt to VR technology, and to voluntarily agree to participate in research. According to accepted criteria, the diagnosis of peripheral unilateral vestibular hypofunction was achieved by responses to bithermal water caloric irrigations, with at least 25% reduced vestibular response on one side when calculated through Jongkees’ formula [20]. On the other hand, for the diagnosis of bilateral vestibular hypofunction, the Barány Society Classification Committee was taken as reference, and the sum of both responses per ear <60/s was accepted as a safe criterion positional tests, ocular-motor tests-smooth pursuit, optokinetic and saccadic tests were evaluated in the VNG test battery. Patients with any physical (neck) discomfort, a history of psychological and/or neurological disorders, central vestibular pathology, and cardiovascular disorders (hypertension, ischemic heart diseases, atherosclerotic coronary artery disease, and so forth) were excluded.

### 2.2. Virtual reality-based VRT intervention

According to the participants included in the study, 11 of the patients experienced right, 10 left, and 4 bilateral VH. The vestibular rehabilitation protocol was prepared with “Danger Ball”, “Beats Saber” and “Shooting Range” balance-based game systems via the ‘Sony PlayStation 4 Virtual Reality’ system for the patients included in the study. Attention, gaze, posture, and hand-eye coordination exercises were included in the VRT protocol (Table 1). VRT program was applied to the patients for 4 weeks, 2 sessions per week, 8 sessions in total. Each session lasted 30–40 min.

**Figure 1:**
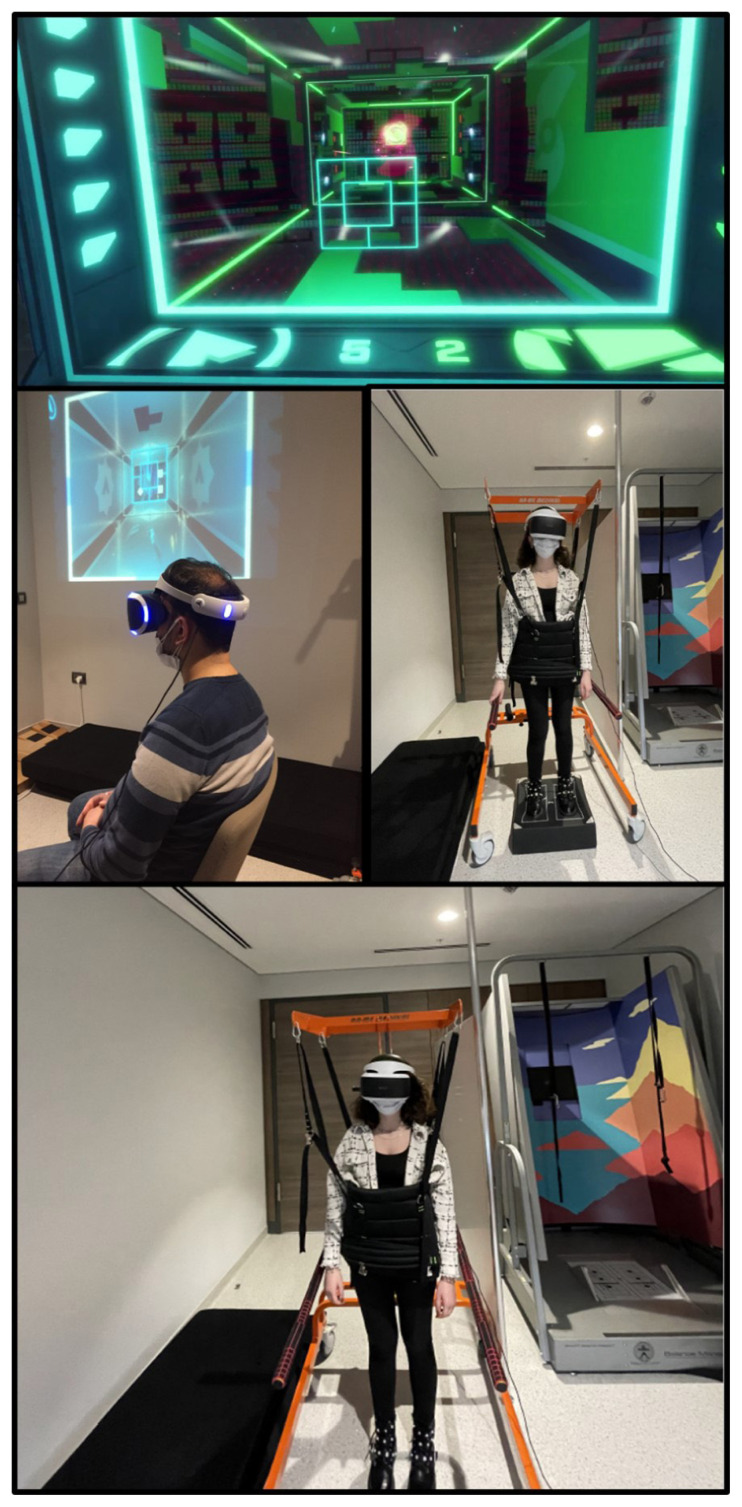
Exercises performed by the patients in sitting, standing, and standing positions on a foam respectively, using the Virtual Reality Worlds Danger Ball game. The patient repeats the manipulated gaze (X1, X2) exercises many times in the game. Thus, it is aimed to improve the patient’s VOR gain. In addition, it is aimed to overcome the balance problems experienced by the patient because of sudden head movements during the day, thanks to the ‘habituation modality’. As of the second week, exercises were started to ensure the active use of the VSR mechanism.

Patients performed a series of exercises that coordinated body movement and shifted it to specified positions, standing 1.5–2 m away from the kinematic camera, which detects movements and maintains the movement area within a specified range. The game systems that were used include exercises that progress gradually from a setting determined according to the patient’s performance and can be passed to different difficulty levels. The clinician supervised the training to ensure correct performance and patient safety. The safety of the patients was ensured by wearing a special vest during the exercises performed on standing and on the foam (Figure 1). Vestibular rehabilitation exercises performed by the patients via VR were followed with the “Epson EB S18” model projection. Thus, the exercise levels were manipulated by evaluating the patients’ feedback on the exercises.

**Figure 2:**
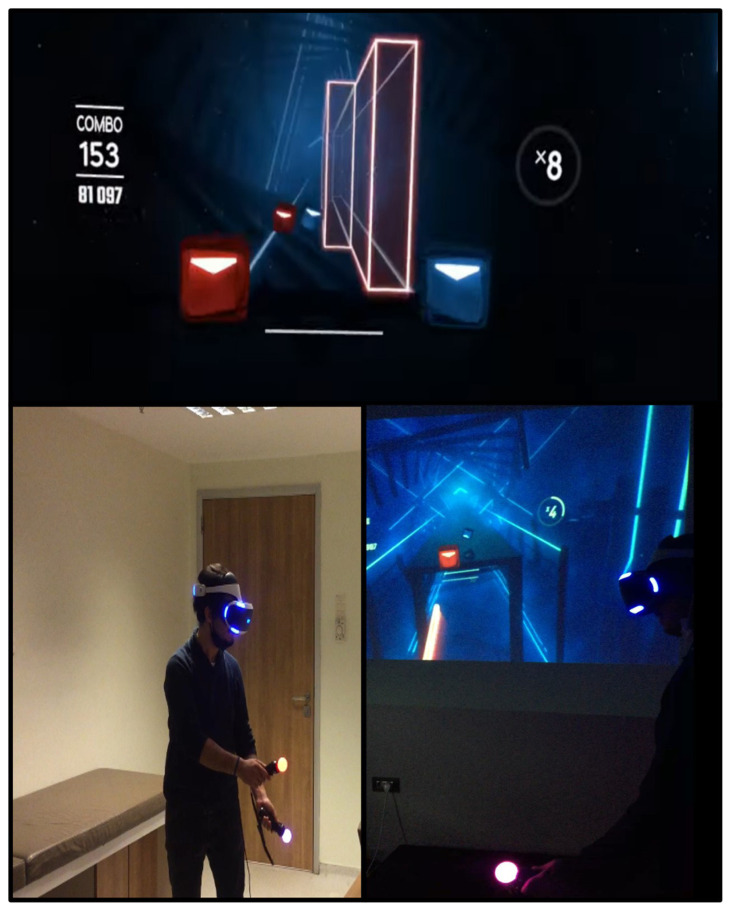
Exercises performed by the patients with the Virtual Reality Worlds Beat Saber game. In this exercise with dual tasks, it is aimed to increase postural control, ensure dynamic balance, correct weight transfer, and develop attention and cognitive skills. In addition, it aims to use the foot and hip ankle strategy in a balanced way for obstacles coming from the right, left, and ahead.

**Figure 3:**
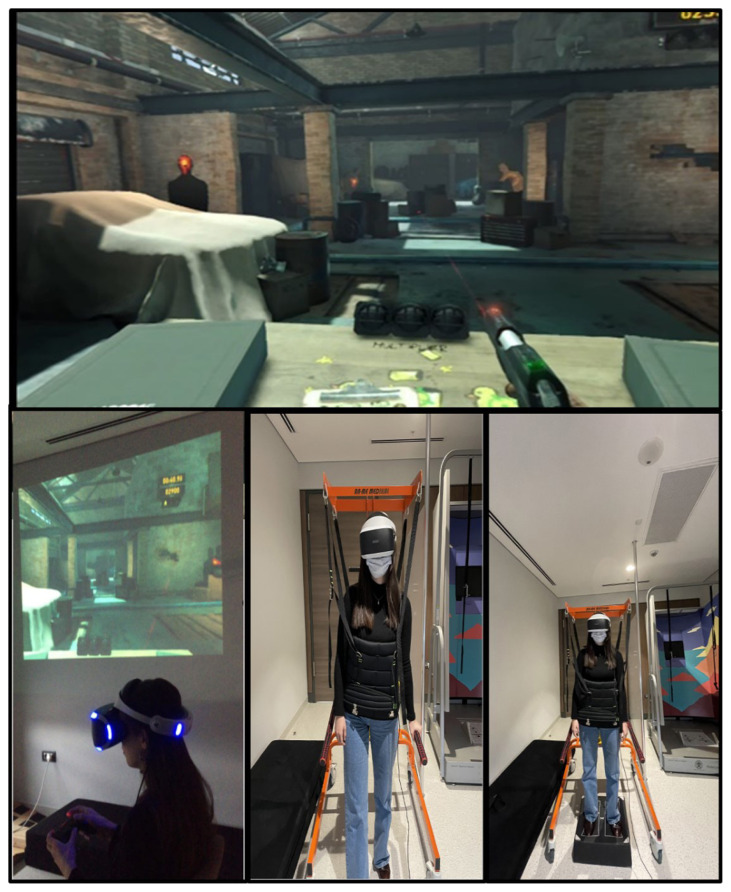
The patients performed saccadic eye movement exercise in VR Shooting Range game (sitting, standing, and standing on foam). It is aimed to improve the VOR gain of the patient through repetitive pursuit and saccadic eye movements. Between the second and fourth weeks, exercises are applied to improve postural stability by increasing the use of somatosensory and visual cues.

The individualized VRT program we prepared is shown in Table 1. The definition, the purpose of balance-based games and how to apply the exercises gradually were detailed in Table 1. In addition, Figure 1 also depicts how the exercises listed in Table 1 were carried out by the patients. Based on the concepts of vestibular ocular reflex (VOR) adaptation and substitution, gaze stability exercises were performed by the patients in the game “Danger Ball”. Gaze stability exercises depend on the supposition that they support vestibular adaptation and involve head movement while maintaining focus on a target, which may be fixed or moving. In the first task (in Danger Ball), patients performed gaze stability exercises focusing on a fixed target and a moving ball in the horizontal and vertical planes (Figure 2). Consecutive points increased the speed of the ball and the difficulty level of the game. Patients gradually performed these exercises from static to dynamic (sitting-standing-standing on a foam). Because it is required to gradually eliminate or replace visual and somatosensory cues in order to educate patients to trust their residual vestibular function, this program advances in this way. Thus, we compelled patients to rely on somatosensory cues and visual cues from the lower extremities during the compensation process (Figure 1 and Figure 3).

The dual-task are involved in Beat Saber exercises. The main purpose of the second exercise is to improve postural stability and the ability to maintain posture in multitasking and patients’ strategy to be able to maintain posture. In the synthetic world created with VR, the patient was given a blue lightsaber in his right hand and a red lightsaber in his left hand. The patients were asked to cut the blue and red boxes that came towards them with a marked arrow, with the same colour lightsaber in their hand. In addition, patients were asked to avoid obstacles coming towards (right-left-ahead) them while performing this task. These exercises were further supplemented with additional auditory stimuli aimed at helping participants make motor exercises more appealing. As the patients successfully complete the task, the level gets more difficult, and the game speed increases. The exercises in this game aimed to improve cognitive functions such as visuospatial and attention and improve alternative motor movements, locomotor control, and postural strategies for postural stability.

The third task (in shooting range), which includes smooth pursuit and saccadic eye movements, aims to improve the retinal error shift that occurs because of the vestibular deficit through substitution exercises. In the task, during active eye-head training between objects, a large eye movement to an objective is made before the head moves to face the target, potentially facilitating the use of preprogrammed eye movements. Thus, the central programming of eye movements was also used to maintain gaze stability. As in the first task, the patients performed the exercises from the static position to the dynamic position in the third task (sitting-standing-standing on a foam) (Figure 3).

### 2.3. Assessments

The patients with PVH were evaluated by computerized dynamic posturography (CDP) with Equitest System version 4.0 equipment, produced by NeuroCom International-USA. The patients were subjected to SOT, ADT, LOS, and RWS, subgroup assessment tests of CDP. Furthermore, patients filled DHI developed by GP Jacobson [21]. DHI is a 25-item measure used to evaluate an individual’s physical, emotional, and functional response to dizziness.

The measure is calculated by summing the scores from each of the 25 questions. Physical subdomain (28 points), emotional subdomain (36 points), and functional subdomain (36 points) scores are also calculated. Higher point totals indicate a higher impact of dizziness on the individual. DHI, SOT, ADT, LOS, and RWS were applied to all patients before and after the VRT program and the 8-week follow-up after VRT. In addition, the Cybersickness Survey was applied to the patients at the end of the VR-based VRT session, which was completed every week.

### 2.4. Data analysis

Statistical analysis of the data in our research was analyzed using the “Statistical Package for the Social Sciences version 22” (SPSS-22) program. The “Kolmogorov–Smirnov Test” was used to assess the normal distribution of variables. “Friedman Test” was used to analyze the patients before, just after, and 8-week follow-up after VRT. The “Wilcoxon Sign Rank Test” was used for pairwise comparison between the groups. The statistical significance level (p-value) was accepted as 0.017 in the Wilcoxon Sign Rank Test results assessed with the Friedman Test, while the p-value was accepted as 0.05.

## 3. Results

All patients had completed the course of treatment and had no reports of side effects pointing to cybersickness symptoms. In Table 2, the demographic information of the participants was indicated.

The values of the parameters of the patients before VRT, after VRT, and at the 8-week follow-up are shown in Table 3. All patients’ values in SOT, DHI, ADT, LOS, and RWS parameters were statistically significant. In addition, pairwise comparisons (time-dependent variables) in which significance was obtained in the test results are shown in Table 4.

**Figure 4:**
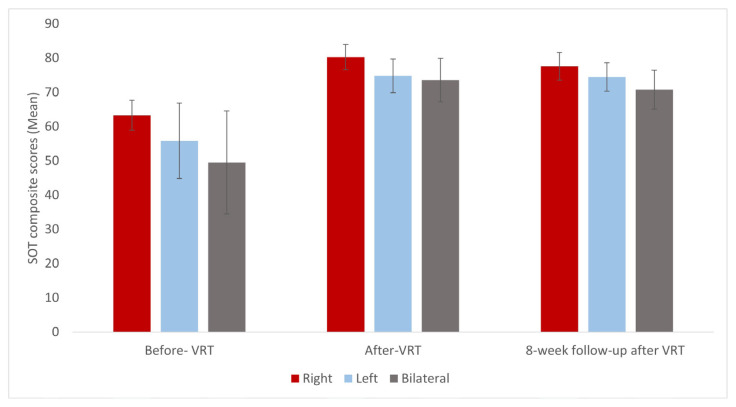
Representation of the mean SOT scores of patients with right, left, and bilateral PVH before, after, and the 8-week follow-up period after VRT.

The patients’ mean SOT composite score showed significant improvement after VRT (p = 0.000). However, there was no statistically significant difference in SOT composite scores at 8-week follow-up after VRT compared to after-VRT (p = 0.372) (Table 3, Table 4). SOT composite scores of patients with PVH before, after, and 8-week follow-up after VRT are shown in Figure 4. SOT composite scores increased after VRT compared with before VRT in all patient groups (Figure 4). However, SOT composite scores at the 8-week follow-up period after VRT decreased compared to after VRT (Figure 4, Table 3).

Patients’ DHI scores showed a drastically decreased after VRT compared to before VRT (p = 0.000) (Table3). In addition, patients’ DHI scores continued to decline in the 8-week follow-up period after VRT compared to after VRT (p = 0.085).

The mean oscillations of the participants in both the ‘toes up’ and ‘toes down’ conditions were statistically decreased after VRT compared to before VRT (p = 0.000, p = 0.000). However, patients’ mean oscillations in both parameters increased in the 8-week follow-up compared with after-VRT but, there were no statistically significant differences (p = 0.518, p = 0.225) (Table 4). In the LOS test, the patients’ reaction time (RT) and movement velocity (MVL) improved after VRT compared to before VRT (p = 0.000). However, there was no statistically significant difference in the values in both RT and MVL in the 8-week follow up after VRT compared to after VRT (p = 0.930, p = 0.230) (Table 3, Table 4). Furthermore, in the LOS test, the patients’ endpoint excursion (EPE), maximum excursion (MXE), and directional control (DC) values showed improvement after VRT compared to before VRT but decreased compared to after VRT in the 8-week follow-up period (Table 3, Table 4).

In the RWS test, the patients’ MVL and DCL values in the horizontal and anteroposterior planes were found to be statistically significant before, after and the 8-week follow-up after VRT (Table 3). Except for MVL values in the anteroposterior plane, all other RWS parameters healed after VRT compared to before VRT (Table 3, Table 4).

## 4. Discussion

This protocol presents the process in which we aimed to investigate whether vestibular rehabilitation using the Sony PlayStation 4® VR system is as effective treatment method in patients with peripheral vestibular loss. Based on the theoretical knowledge of that feedback and practice improve motor learning, incorporating feedback technology such as virtual reality in rehabilitation programs presents clinicians with considerable opportunities to improve patient outcomes [22].

Uğur and Konukseven utilized vestibular rehabilitation in patients with motion sickness using Sony PlayStation 4 VR. They reported Sony PlayStation 4 VR as an effective, useful, entertaining, adaptive, and motivational option for the treatment of motion sickness patients [23]. Also, Lee et al. utilized game exercises with Sony PlayStation® on a 27-year-old stroke patient [24]. The trainings lasted for 30 min, 3 days a week, for a total of 18 sessions. The results showed that the patient got 34 points from the Motor Evaluation Scale, 14 points more than the preevaluation point; the patient got 48 points from the Berg Balance Scale, 16 points more than the preevaluation score. This case report demonstrated that training utilizing Sony PlayStation video games may be an effective method to improve gait and balance in a young patient [24]. Sony PlayStation, which is primarily used for gaming and entertainment, has also been used in the literature for potential therapeutic purposes for vestibular patients. In this study, we created a customized vestibular rehabilitation protocol utilizing this equipment and used it therapeutically for patients with PVH. The cumulative exposure protocol was 240 min per patient in our current study. Bergeron et al. in their meta-analysis of VR techniques of VRT in patients suffering from the vertigo of peripheral origin emphasized that VR treatment should last at least 150 min of cumulated virtual environment exposure to achieve positive outcomes [19]. They stated that neither the duration of a particular session nor the total number of sessions is a predictive outcome factor. A systemic review by Bergeron et al. expressed concern that the use of virtual reality might be limited by motion sickness or cybersickness because of excessive sensory stimulation [19]. None of the patients who completed training complained of cybersickness or exacerbating symptoms during rehabilitation.

Meldrum et al. compared VR-based vestibular rehabilitation with conventional VRT methods in the literature. They revealed that vestibular rehabilitation with Nintendo Wii Fit plus VR is much more effective in unilateral vestibular pathologies [25]. Rosiak et al. showed that hybrid VR-based systems effectively reduce symptoms in patients with PVH [13]. Sparrer et al. compared selected traditional vestibular rehabilitation methods and a VR group. The SOT values of both groups on the fifth day and ten weeks after rehabilitation treatment were compared. SOT values of the group that received VR-based vestibular rehabilitation programs were found to be improved positively compared to the other group [26]. In another study, Meldrum et al. divided the treatment programs of individuals with vestibular dysfunction into two randomized groups as traditional vestibular rehabilitation and virtual reality systems. SOT values improved in both groups, and there was no statistically significant difference [26]. In our study, the SOT values of our patients increased by 20.33 points at the end of the 4-week training, while the results of the 8-week follow-up showed an increase of 18.96 points relative to their first score (p < 0.017).

Viziano et al. investigated VR-based vestibular rehabilitation therapy for a long period of time (twelve months) in patients with unilateral PVH. The study revealed that the DHI scores of the patients who had a 20-min session once a month decreased statistically significantly at the end of the year [27]. In another study, Stankiewicz et al. applied a vestibular rehabilitation program integrated with VR technology to patients diagnosed with unilateral vestibular hypofunction. They showed that the scores obtained by the patients on the “Vertigo Symptom Scale (VSS)” decreased by eight points compared to the prerehabilitation, and a statistically significant difference was obtained [28]. Jiao et al. reported that after a 12-week VR-based VRT program in vestibular patients, the patients’ VSS scores decreased, and a statistically significant difference was obtained (p < 0.01) [29]. Coelho et al. revealed that DHI scores decreased from 56 points to 25 at the end of twelve sessions in six weeks, with two sessions per week. Furthermore, the scores decreased from 25 points to 19 in the third-month follow-up [30]. In our study, while the patients’ DHI scores decreased from 54 to 19 after VRT, it showed a further decrease from 19 to 16 in the 8-week follow-up. It has been subjectively revealed that the patients’ symptoms have decreased, and their quality of life has improved.

Phillips et al. performed VR-based VRT via the “ski slalom” game to 40 patients with dizziness with a DHI score above 20 for 16 weeks. The virtual game aimed to enable the patient to control the posture, transfer the centre of gravity to the right and left, and use the hip-knee-ankle strategies correctly and in a balanced way. At the end of sixteen weeks, patients’ balance functions improved, and their DHI score decreased significantly [31]. Miziara et al., on the other hand, applied vestibular rehabilitation to BPPV patients using the “ski slalom” game and “tightrope walking VR game systems”, consisting of two sessions and 30 min per week for 4 weeks. Researchers have shown that individuals’ DHI scores and postural oscillations decreased significantly after 4 weeks [32]. In our VRT protocol, the game called ‘Beat Saber’ aims to use the foot and hip ankle strategy in a balanced way for obstacles coming from the right, left, and ahead. Thus, we made sure the patient to transferred their weight correctly and use the foot-hip-ankle strategy properly and in a balanced way when bending over. The parameters in the RWS test in our study showed significant improvement for the patients after rehabilitation. In the 8-week follow-up after VRT, although DCL and MVL decreased, there was no significant difference compared to pre-VRT values.

Studies conducted in the last two decades have revealed that vestibular system diseases are not only related to the balance system but also play an active role in cognitive processes [33]. The literature emphasizes that the imbalance problem of patients with unilateral vestibular loss is not only related to peripheral vestibular disease. Essentially, a dysfunction involving the vestibular nucleus, limbic system, and cortical areas can affect both cognition and spatial orientation in the process [34]. Numerous studies in animals and humans have shown that vestibular dysfunction is associated with various forms of cognitive impairment [35,36]. One of the first studies on cognitive function in patients with vestibular disorders was Grimm et al.’s study reporting perceptual and memory deficits in patients with perilymph fistula syndrome, with more than 85% of patients stating memory loss [36]. In a study conducted with animals published in 2015, bilateral vestibular lesions were shown to cause severe spatial memory impairment [35]. While another study in 2007 reported that the left hippocampal volume was smaller in people with Meniere’s disease than in healthy individuals [37–39].

Redfern et al. investigated the cognitive effects on postural control of 15 patients with unilateral vestibular loss in four different conditions [39]. They made the participants sit and stand on a fixed floor and a fixed visual environment, standing on a moving floor with a fixed visual environment, and a pseudorandom translation with a fixed visual environment. The patients were assigned the simple auditory reaction time task and the auditory selection reaction time task. Reaction times were significantly longer for patients in all tasks compared to the control group. Moreover, it was observed that the difference increased with the complexity of the task. However, it was seen that the patients’ reaction times were longer even when sitting, and the reaction time was increased for more complex cognitive tasks. Thus, although vestibular compensation was formed (no complaints of dizziness imbalance), the reaction time was still prolonged in patients with unilateral vestibular loss [39]. In our research, using the virtual game called “Beat Saber”, the patients were asked to cut boxes of a certain colour with the same-coloured lightsabers in their hands, in the direction of the arrows on the boxes. While cutting the boxes in certain shapes, the patient tried to maintain balance by simultaneously bending to the right, the left, and leaning against the walls. Our goal was for the patient to correctly complete both attention and weight transfer exercises in this dual-task module. It is interpreted that the attention exercises contribute to the patients’ acceleration of the dynamic compensation process in the reduction of the reaction times (in the LOS test), the increase in the speed of movement and the development of directional control.

### 4.1. Limitations of the study

The VRT protocol we created in our study was prepared by selecting certain virtual reality games available based on the American Physical Therapy Association Neurology Section: Vestibular Rehabilitation for Peripheral Vestibular Hypofunction: An Evidence-Based Clinical Practice Guideline. Thus, this protocol offered patients the opportunity to recover in a pleasant and safe environment with an evidence-based approach that could meet the needs of patients.

As a limitation, in this study, the patients’ ability to maintain LOS, SOT (somatosensory, visual, and vestibular sensory analysis), and posture were evaluated, but their VOR could not be evaluated objectively. Therefore, patients’ VOR responses in this study could not be correlated with data from CDP (DHI, SOT, or LOS, et al.).

Furthermore, future studies may need to standardize the VR-based VRT protocol with standard equipment and contents. Additionally, it may be necessary to document more about cyber sickness and its effects as an effect of VR use (a validated and reliable cybersickness questionnaire).

Few studies are found in the literature showing the efficacy of VR-based VRT in patients with vestibular dysfunction in the mid-term. In our study, we showed that the symptoms of PVH patients were reduced, and their quality of life improved after VR-based VRT treatment. It was also observed that the DHI scores of the patients decreased in the 8-week follow-up period, and the values in the CDP subtest groups were generally stable after VRT. Therefore, VR-based VRT we created has shown that it remains effective in people diagnosed with PVH in the mid-term.

## Figures and Tables

**Table 1:** Virtual reality-based VRT protocol created in our research.

Exercise type	Description of the E-exercise	Purpose of the exercise	The practice program of the exercises (twice a week [×2])
**Virtual Reality Worlds Danger Ball**	In the first stage, the patient follows the ball hitting the plates by providing eye-head coordination and tries to get point. In the second stage, the patient tries to score points by sending the ball to the area protected by opponent. This exercise aims to increase the decreased vestibular responses thanks to head movements.	The patient repeats the manipulated gaze (X1, X2) exercises many times in the game. Thus, it is aimed to improve the patient’s VOR gain. In addition, it is aimed to overcome the balance problems experienced by the patient because of sudden head movements during the day, thanks to the ‘habituation modality’. As of the second week, exercises were started to ensure the active use of the VSR mechanism.	**First week**, the patient performs the exercises at an ‘easy’ level in the sitting position. **Second week**, the patient performs the exercises at an ‘easy’ level in the standing position. **Third week**, the patient performs the exercises at a ‘medium’ level in the standing position. **Fourth week**, the patient performs “medium-hard” level exercises standing on the foam.
**Virtual Reality Worlds Beat Saber**	The patient tries to cut the red and blue boxes in the direction of the arrows with a red lightsaber in his left hand and a blue lightsaber in his right hand. In the meantime, the patient tries to do these procedures by avoiding the walls that come towards him randomly.	In this exercise with dual tasks, it is aimed to increase postural control, ensure dynamic balance, correct weight transfer, and develop attention and cognitive skills. In addition, it aims to use the foot and hip ankle strategy in a balanced way for obstacles coming from the right, left, and ahead.	**First week** the patient performs the exercises at an ‘easy’ level in the standing position. **Second week**, the patient performs the exercises at a ‘medium’ level in the standing position. **Third week**, the patient performs the exercises at a ‘hard’ level in the standing position. **Fourth week**, the patient performs “hard” level in the standing position.
**Virtual Reality Worlds Shooting Range**	The patient follows the random targets with her/his eyes while the head is fixed and tries to shoot them with the VR move controller.	It is aimed to improve the VOR gain of the patient through repetitive pursuit and saccadic eye movements. Between the second and fourth weeks, exercises are applied to improve postural stability by increasing use of somatosensory and visual cues.	**First week**, the patient performs the exercises at an ‘easy’ level in the sitting position. **Second week**, the patient performs the exercises at a ‘medium’ level in the standing position. **Third week**, the patient performs the exercises at a ‘hard’ level in the standing position. **Fourth week**, the patient performs the exercises at a ‘hard’ level in the standing on the foam position.

**Table 2:** Sociodemographic information of the participants.

Sex	N	Age Mean ± SD (min-max)	Affected side in patients diagnosed with PVH		
			Right	Left	Bilateral
Female	15	43.33 ± 13.12 (18–65)	6	6	2
Male	10	46.00 ± 8.74 (32–60)	5	4	2
Total	25	44.4 ± 11.44 (18–68)	11	10	4
**Diagnosis**			**Participants (%)**		
Vestibular neuritis			13(52)		
Meniere’s disease			7(28)		
Labyrinthectomy			2(8)		
Vestibulotoxicity			1(4)		
**Diagnosis made by:**					
% caloric weakness right side			56.63 ± 24.42		
% caloric weakness left side			52.37 ± 22.59		
**Months since first onset of symptoms**			13.36 ± 5.71		
**Clinical diagnosis; positive head thrust test or presence of head-shaking nystagmus**					
Present			10(40)		
Absent			15(60)		
**Cerebellar test**					
Normal			25(100)		
Abnormal			0 (0)		
**Fall history**					
None			16(64)		
One			7(28)		
More than one			2(8)		

N: Participants, SD: Standard deviation, Min: Minimum, Max: Maximum, PVH: Peripheral vestibular hypofunction

NOTE. Values are mean ± SD, n, or n (%).

**Table 3:** Parameter values of the patients before, after, and the 8-week follow-up period after VRT.

Parameter	Before VRT Mean ± SD (min-max)	After VRT Mean ± SD (min-max)	8-week follow-up after VRT Mean ± SD (min-max)	p-value (Friedman Test)
SOT	58.08 ± 10.42 (29–70)	77.16 ± 5.2 (67–86)	76.40 ± 5.41 (65–86)	**p = 0.000** ^*^
DHI	54.60 ± 17.85 (30–88)	19.20 ± 13.26 (4–44)	16.84 ± 11.55 (0–36)	**p = 0.000** ^*^
ADT toes up	68.60 ± 15.66 (44.0–110.2)	55.64 ± 11.56 (32.60–84.2)	58.0 ± 8.38 (31.60–33.4)	**p = 0.000** ^*^
ADT toes down	49.65 ± 10.85 (29.2–75.0)	40.68 ± 6.51 (27.6–55.0)	43.18 ± 7.62 (26.2–58.8)	**p = 0.000** ^*^
LOS RT	1.82 ± 0.8 (0.9–4.2)	0.93 ± 0.26 (0.48–1.4)	0.90 ± 0.18 (0.60–1.3)	**p = 0.000** ^*^
LOS MVL	2.69 ± 0.9 (1.30–5.0)	4.0 ± 1.05 (2.20–6.4)	3.84 ± 0.83 (2.80–5.3)	**p = 0.000** ^*^
LOS EPE	63.92 ± 5.9 (52–73)	73.52 ± 6.0 (64–85)	66.72 ± 3.42 (61–72)	**p = 0.000** ^*^
LOS MXE	76.92 ± 5.61 (67–90)	86.24 ± 5.87 (73–96)	81.60 ± 6.22 (72–98)	**p = 0.000** ^*^
LOS DCL	69.84 ± 6.49 (59 ± 82)	78.52 ± 6.14 (64–89)	77.84 ± 3.81 (68–85)	**p = 0.000** ^*^
Horizontal plane RWS MVL	4.27 ± 1.49 (1.70–8.40)	5.18 ± 0.9 (3.70–6.9)	4.49 ± 0.79 (2.90–6.10)	**p = 0.000** ^*^
Horizontal plane RWS DCL	78.84 ± 5.18 (71–90)	84.60 ± 4.65 (75–92)	80.12 ± 4.23 (70–87)	**p = 0.001** ^*^
Anteroposterior plane RWS MVL	3.08 ± 0.91 (1.1–4.31)	3.86 ± 0.84 (2.0–5.4)	4.15 ± 0.76 (2.8–5.8)	**p = 0.000** ^*^
Anteroposterior plane RWS DCL	70.72 ± 9.36 (50–86)	80.6 ± 7.29 (62–94)	77.72 ± 5.82 (65–87)	**p = 0.000** ^*^

SD: Standard deviation. Min: Minimum, Max: Maximum. SOT: Sensory Organization Test, DHI: Dizziness Handicap Inventory, ADT: Adaptation Test. LOS: Limits of stability. RT: Reaction time. MVL: Movement velocity, EPE: Endpoint excursion, MXE: Maximum excursion, DCL: Directional control, RWS: Rhythmic weight shift)

Friedman Test p < 0.05^*^

**Table 4:** Paired comparison between groups.

Parameter	Before VRT-after VRT z p-value	After VRT-8-week follow up z p-value	Before VRT-8-week follow up z p-value
SOT	−4.37	−0.89	−4.37
	**p = 0.000** ^*^	p = 0.372	**p = 0.000** ^*^
DHI	−4.37	−1.72	−4.37
	**p = 0.000** ^*^	p = 0.085	**p = 0.000** ^*^
ADT toes up	−3.64	−0.64	−3.17
	**p = 0.000** ^*^	p = 0.518	**p = 0.001** ^*^
ADT toes down	−3.60	−1.21	−2.72
	**p = 0.000** ^*^	p = 0.225	**p = 0.009** ^*^
LOS RT	−4.29	−0.88	−4.37
	**p = 0.000** ^*^	p = 0.930	**p = 0.000** ^*^
LOS MVL	−4.11	−1.27	−4.28
	**p = 0.000** ^*^	p = 0.203	**p = 0.000** ^*^
LOS EPE	−4.33	−4.10	−2.23
	**p = 0.000** ^*^	**p = 0.000** ^*^	p = 0.026
LOS MXE	−4.04	−3.50	−2.96
	**p = 0.000** ^*^	**p = 0.000** ^*^	**p = 0.003** ^*^
LOS DCL	−4.03	−0.82	−3.91
	**p = 0.000** ^*^	p = 0.411	**p = 0.000** ^*^
Horizontal plane RWS MVL	−2.99	−3.73	−1.40
	**p = 0.003** ^*^	**p = 0.000** ^*^	p = 0.161
Horizontal plane RWS DCL	−3.61	−3.51	−0.97
	**p = 0.000** ^*^	**p = 0.000** ^*^	p = 0.330
Anteroposterior plane RWS MVL	−3.59	−2.24	−3.94
	**p = 0.000** ^*^	p = 0.026	**p = 0.000** ^*^
Anteroposterior plane RWS DCL	−4.18	−2.27	−2.29
	**p = 0.000** ^*^	p = 0.023	**p = 0.004** ^*^

SOT: Sensory Organization Test, DHI: Dizziness Handicap Inventory, ADT: Adaptation Test, LOS: Limits of stability, RT: Reaction time, MVL: Movement velocity, EPE: Endpoint excursion, MXE: Maximum excursion, DCL: Directional control, RWS: Rhythmic weight shift)

Wilcoxon Sign Rank p < 0.017^*^
